# Knowledge mapping visualization of the pulmonary ground-glass opacity published in the web of science

**DOI:** 10.3389/fonc.2022.1075350

**Published:** 2022-12-22

**Authors:** Xingchen Li, Guochao Zhang, Shugeng Gao, Qi Xue, Jie He

**Affiliations:** Department of Thoracic Surgery, National Cancer Center/National Clinical Research Center for Cancer/Cancer Hospital, Chinese Academy of Medical Sciences and Peking Union Medical College, Beijing, China

**Keywords:** bibliometric analysis, pulmonary ground-glass opacity, lung cancer, visualization, CT

## Abstract

**Objectives:**

With low-dose computed tomography(CT) lung cancer screening, many studies with an increasing number of patients with ground-glass opacity (GGO) are published. Hence, the present study aimed to analyze the published studies on GGO using bibliometric analysis. The findings could provide a basis for future research in GGO and for understanding past advances and trends in the field.

**Methods:**

Published studies on GGO were obtained from the Web of Science Core Collection. A bibliometric analysis was conducted using the R package and VOSviewer for countries, institutions, journals, authors, keywords, and articles relevant to GGO. In addition, a bibliometric map was created to visualize the relationship.

**Results:**

The number of publications on GGO has been increasing since 2011. China is ranked as the most prolific country; however, Japan has the highest number of citations for its published articles. Seoul National University and Professor Jin Mo Goo from Korea had the highest publications. Most top 10 journals specialized in the field of lung diseases. Radiology is a comprehensive journal with the greatest number of citations and highest H-index than other journals. Using bibliometric analysis, research topics on “prognosis and diagnosis,” “artificial intelligence,” “treatment,” “preoperative positioning and minimally invasive surgery,” and “pathology of GGO” were identified. Artificial intelligence diagnosis and minimally invasive treatment may be the future of GGO. In addition, most top 10 literatures in this field were guidelines for lung cancer and pulmonary nodules.

**Conclusions:**

The publication volume of GGO has increased rapidly. The top three countries with the highest number of published articles were China, Japan, and the United States. Japan had the most significant number of citations for published articles. Most key journals specialized in the field of lung diseases. Artificial intelligence diagnosis and minimally invasive treatment may be the future of GGO.

## 1 Introduction

Lung cancer is the primary cause of cancer-related deaths globally, affecting 24% and 23% of men and women, respectively (1). The most common pathological subtype of lung cancer is lung adenocarcinoma.

Patients are commonly diagnosed at local advanced or late stages, resulting in poor prognosis. Hence, early detection and treatment of lung cancer may improve overall survival rates. With low-dose CT lung cancer screening, the National Lung Screening Trial showed reduced lung cancer-related mortality (2). Currently, low-dose spiral CT is a vital tool in lung cancer screening (3). However, ground glass opacity (GGO) with low-dose CT has become increasingly common.

GGOs are focal, round or irregular in shape, with densely enlivened opacities <3 cm diameter surrounded by lung parenchyma (4). Most GGOs are benign, but some may be early forms of lung cancer (5). In recent years, the development of methods for accurately predicting the nature of GGO and prescribing the appropriate treatment are increasingly investigated. In addition, many studies and books are published in this field.

Due to a large number of publications and significant variations in quality, each article requires a scientific method to determine its significance. Identifying valuable information from the increasing number of publications is a great challenge for scientists. As a mathematical and statistical method, the bibliometric analysis estimates the influence or impact of the research articles (6, 7). Bibliometrics has been widely used to analyze the literature on lung cancer and lung nodules (8–10). So far, a bibliometric analysis of the literature in this field has not been reported. Hence, bibliometric analysis was conducted in this study to summarize the foundation for and development of GGO and provide summaries of current research progress, hotspots, and next steps for GGO. These findings may provide a better understanding of GGO for future studies.

## 2 Methods

### 2.1 Search strategies and analysis tools

Two independent authors searched for relevant publications on the Web of Science (http://webofscience.com; Thomson Reuters, Toronto, Canada). The data were collected from the Web of Science Core Collection (WoSCC) database’s establishment to December 31, 2021. The WoSCC database was selected for three reasons: it is commonly used in bibliometric analysis, it can provide comprehensive information to the bibliometric software, and it is considered one of the most influential databases. Lung cancer, ground glass nodule, and GGO were used as search terms, with their relevant synonyms or abbreviations. The detailed search strategy is shown in **Supplementary Figure S1**. The study included only articles and reviews in the English language. The literature screening process is shown in **Supplementary Figure S2**. The present study did not require ethical approval as the data were retrospectively downloaded from the database.

The R package bibliometrix was used to conduct quantitative research and assess the scientific impact of the research. Bibliometrix provides tools to perform a complete bibliometric analysis following the Science Mapping Workflow (11). In addition, scholars use the tool to publish extensive bibliometric literature. Moreover, the bibliometric maps were created using VOSviewer. The VOSviewer uses a similarity measurement to compute the related strength between data elements (12). In VOSviewer maps, the size of the label/circle correlates to the weight of the node: the stronger the weight, the larger the label and circle. The line connecting two nodes represents the strength of association. The frequency of co-occurring phrases between the nodes constructs a cluster of closely linked nodes.

### 2.2 Field analyses

Statistical analysis was performed on the following fields: title, keywords, author, author’s institution, author’s country or region, journal name, total citations, and average annual citations. A statistical survey of the annual number of articles published was conducted to observe the development of the discipline. The top 10 active authors, journals, funding agencies, and countries were listed to define important scholars and institutions.

To some extent, the number of citations constitutes the importance of the study. Hence, a statistical analysis was conducted on the authors, author’s country or region, author’s institution, and journal according to the number of citations to determine their importance in the field. The H-index measures productivity and citations at the author level (13).It provides a more comprehensive assessment of the scholar’s work compared to other quantitative measures (14). In addition, the H-index of journals was calculated to identify the most influential journals in the field. Furthermore, the minimum keyword frequency was set to 7 times in VOSviewer for keyword co-occurrence analysis.

## 3 Results

### 3.1 Publication literature overview

**Figure 1:**
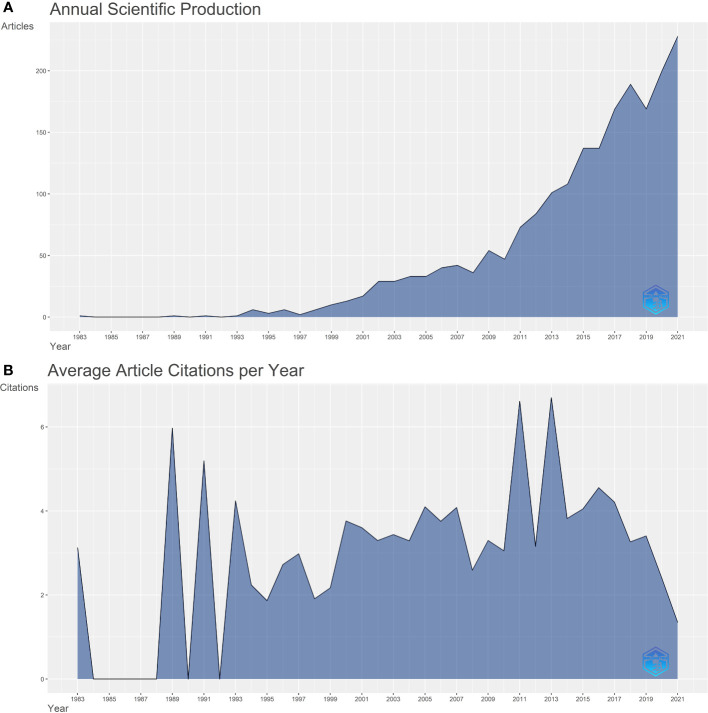
**(A)** A line chart of the number of publications published each year, 1983-2021. **(B)** A line chart showing the average number of citations per article per year.

In the present study, 2,005 publications were reviewed. Of these, 1824 were original articles and 181 were reviews. As shown in **Figure 1A**, the number of publications before 2010 was relatively small; however, the number of publications has since rapidly grown. The average annual growth rate of the number of publications is 19.4%. The average annual number of citations in the field was plotted to determine the significant published literature. As shown in **Figure 1B**, the number of literature citations in 2011 and 2013 were relatively high, suggesting that literature in this period had an important impact on the discipline.

### 3.2 Country and regional analyses

**Figure 2:**
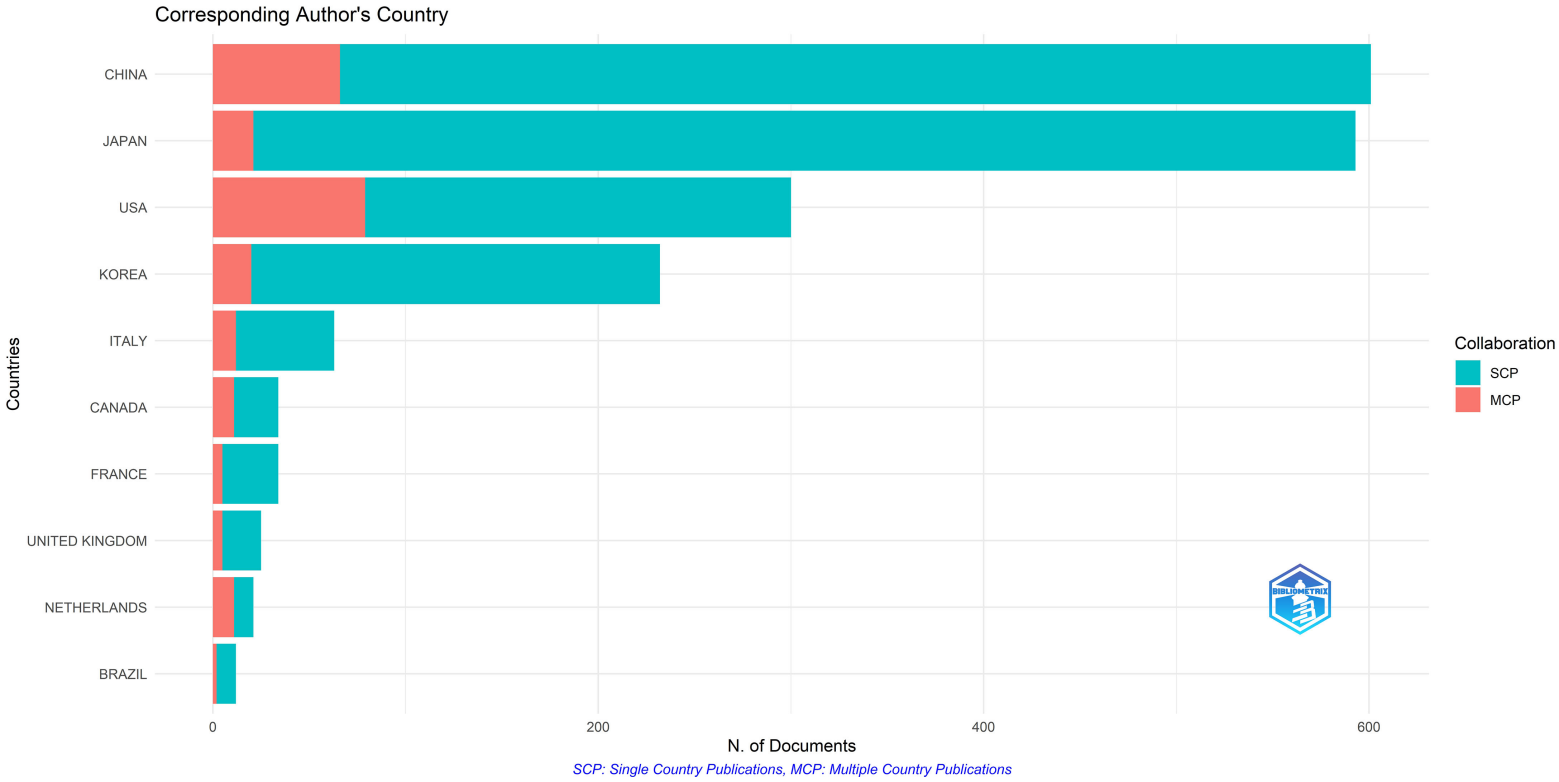
Corresponding author’s country.

China, Japan, the United States, South Korea, and Italy showed the highest number of published articles. Most research in this area is single-country research, with a smaller proportion of collaborative research conducted in multiple countries (**Figure 2**). Although China had the largest number of publications, Japan had a significant total number of published citations at 17,571. All countries in this field had cooperative relationships (**Supplementary Figure S3**). The wider the line among countries or regions, the closer their cooperation. The findings reported less cooperation among countries in this field of research. The United States has more collaborative research with other countries, including China, Japan, and Korea.

### 3.3 Active institutions and authors

**Figure 3:**
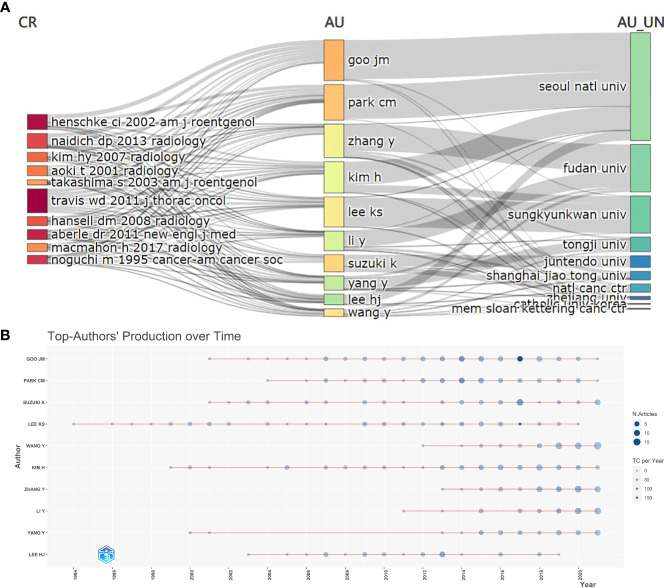
**(A)** The top 10 most published authors. CR: Source Journal. AU: Author. AU-UN: Author University. **(B)** Top-authors’ production over time.

The top 10 authors and institutions were analyzed and listed based on the number of publications to identify the key authors and research institutions in this field (**Figure 3A**). Among them, Seoul National University and Professor Jin Mo Goo from Korea had the highest publications. Seoul National University contributed to 453 articles, and Jin Mo Goo published 73 articles, accounting for 13% of the articles in this field. A contribution timeline of the top 10 authors in this field is shown in **Figure 3B**. Professor Jin Mo Goo was the top author based on the number of publications; however, his publications have reduced in recent years. Professor Suzuki K of Juntendo University, Japan, Professor Wang Y of Fudan University, and Professor Kai Zhang of Fudan University had significantly high publication volumes in the last 3 years.

### 3.4 Key journals

Based on the number of published articles, the top 10 magazines were listed. In addition, the total number of citations and H-index were included for comprehensively assessing the influence of journals. As shown in **Supplementary Figure S4**, most of the top 10 journals specialized in the field of lung diseases. Among these, the Radiology journal had a significant number of citations and the highest H-index. The Journal of Thoracic Disease and European Radiology published more papers than other journals in this field.

### 3.5 Keyword analysis

**Figure 4:**
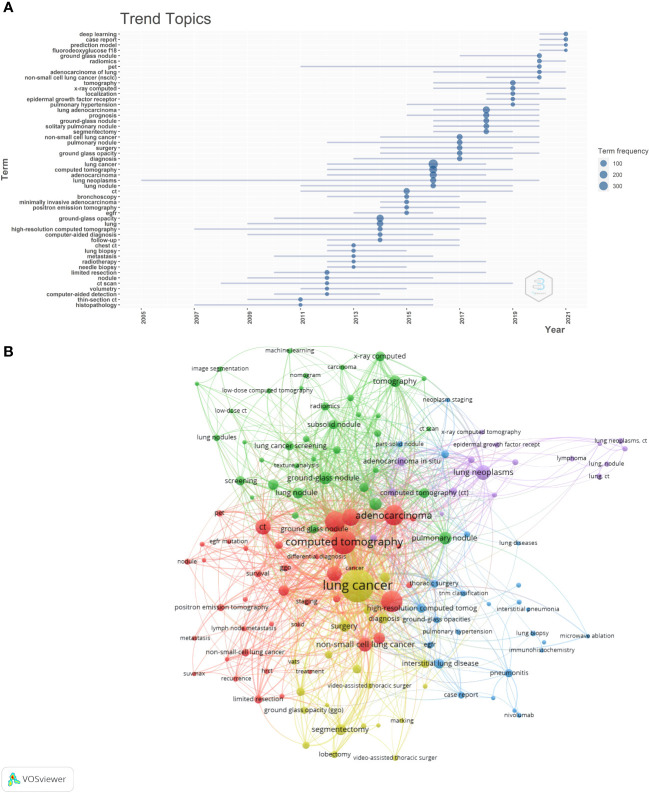
**(A)** The graph shows the change over time of the most frequent keywords in each year, with the size of the blue nodes representing the frequency of keyword occurrences. **(B)** This is the cluster analysis map of keywords. The size of the dot represents the frequency of keyword occurrence, and the color of the dot represents the keyword clustering.

Keywords help observe not only the correlation among the research topics but also the direction of the field. First, the frequency of keywords in the last 10 years was plotted based on the frequency of author keywords (**Figure 4A**). The main research hotspots of GGO between 2011 and 2015 were chest CT, high-resolution CT, limited resection, and lung biopsy. Essential research keywords, including deep learning and radiomics, positron emission tomography(PET), and epidermal growth factor receptor, have become increasingly popular in the past 3 years. A total of 141 keywords were identified using the keyword co-occurrence analysis. The keyword co-occurrence network and density maps are shown in **Figure 4B**. In addition, five clusters were identified. Based on the density map, lung cancer, adenocarcinoma, and CT examination were the most studied topics in this research area.

As shown in **Figure 4B**, the color of each node represents a cluster, and the size of the node represents the frequency of the keyword. Cluster 1 (red), the largest one, had 39 co-occurring words, including adenocarcinoma, CT, high resolution computed tomography(HRCT), PET/CT, and survival. These are prognosis and diagnosis topics. Cluster 2 (green) had 37 terms, such as artificial intelligence, classification, lung adenocarcinoma, diagnostic imaging, radiomics, and texture analysis. Cluster 3 (blue) contained 25 keywords related to treatment, including GGO, immunohistochemistry, microwave ablation, pembrolizumab, radiotherapy, thoracic surgery, and TNM classification. Cluster 4 (yellow) included 21 co-occurring words, such as diagnosis, lobectomy, marking, sublobar resection, and video-assisted thoracic surgery. The theme of this cluster was preoperative positioning and minimally invasive surgery. Cluster 5 (purple) was the last cluster with 17 co-occurring words, including adenocarcinoma in situ, atypical adenomatous hyperplasia, bronchioloalveolar cell carcinoma, GGO, and toxicity, which were associated with GGO pathology.

### 3.6 Most cited papers

The essential literatures in this field were identified, listing the 10 most cited papers (**Table 1**). Important literature in this field was published between 2011 and 2018. Particularly, significant numbers of important literature were published from 2011 to 2013. Most top 10 crucial literature in this field were guidelines for lung cancer and pulmonary nodules. However, since guideline formulation requires a large sample of randomized controlled trials as evidence, key randomized controlled trials also be published during the corresponding period.

**Table 1:** The top ten articles with the most total citations.

Paper	DOI	Total Citations	TC per Year
TRAVIS WD, 2011, J THORAC ONCOL	10.1097/JTO.0b013e318206a221	3043	253.5833
MACMAHON H, 2017, RADIOLOGY	10.1148/radiol.2017161659	895	149.1667
HOWINGTON JA, 2013, CHEST	10.1378/chest.12-2359	711	71.1
GOULD MK, 2013, CHEST	10.1378/chest.12-2351	680	68
NAIDICH DP, 2013, RADIOLOGY	10.1148/radiol.12120628	653	65.3
TRAVIS WD, 2016, J THORAC ONCOL	10.1016/j.jtho.2016.03.025	340	48.5714
WOOD DE, 2018, J NATL COMPR CANC NE	10.6004/jnccn.2018.0020	229	45.8
CALLISTER MEJ, 2015, THORAX	10.1136/thoraxjnl-2015-207168	251	31.375
SUZUKI K, 2011, J THORAC ONCOL	10.1097/JTO.0b013e31821038ab	336	28
DELAUNAY M, 2017, EUR RESPIR J	10.1183/13993003.00050-2017	165	27.5

## 4 Discussion

In the present study, bibliometric and professional analyses were performed to identify the key knowledge and developmental trends on GGO. According to the bibliometric analysis of publication time, an increased number of scientific articles are published every year, possibly because GGO has become increasingly common in clinical practices due to CT examinations. Hence, GGO is gaining the attention of many scholars.

The highest average numbers of citations per year were recorded in 2011 and 2013, with significant published articles during this period. The literature citation analysis showed that the key guidelines in this field were published in 2011 and 2013. For example, the article “International association for the study of lung cancer/American thoracic society/European respiratory society international multidisciplinary classification of lung adenocarcinoma” published in 2011, defined the pathological types of lung cancer (15). The article “Recommendations for the management of subsolid pulmonary nodules detected at CT: a statement from the Fleischner Society,” published in 2013, standardized the diagnosis and treatment process after CT-found pulmonary nodules (16).

China, Japan, the United States, and South Korea were the top countries with the most published articles. Although China had a significant number of publications compared to others, Japan had the highest number of citations for published articles at 17,571. This suggests that Chinese articles need quality improvement. The cooperative relationships among all countries in this field are shown in **Supplementary Figure S3**. The findings showed that the United States had more collaborative research with other countries than other countries, particularly with China, Japan, and Korea.

Seoul National University and Professor Jin Mo Goo from Korea had the highest publications. Professor Jin Mo Goo primarily focused on GGO diagnosis and pathological classification (17–19), suggesting that diagnosis and pathological typing of GGO are crucial in research. Professors Suzuki K of Juntendo University, Japan, and Wang Y of Fudan University, China, showed higher publication volume in recent years (**Figure 3B**). The research content of an author with significant recent publications may reflect the recent research directions in this field. In the last 2 years, Professor Suzuki K published articles on surgical treatment and prognosis of GGO. In addition, Professor Wang Y primarily researches artificial intelligence-based diagnosis of pulmonary nodules (20, 21).

The core journals in this field can be understood better by quantitatively analyzing their bibliographic information. In **Supplementary Figure S4**, most top 10 journals specialized in lung diseases. However, Radiology was the comprehensive journal with the highest number of citations and H-index. Moreover, the field of study of a journal may change over time. Journal of Thoracic Disease and European Radiology published many papers in this area over the past 3 years. The latest research advances in this field can be found in leading journals.

Chest CT, high-resolution CT, limited resections, and lung biopsies were the primary research hotspots of GGO between 2011 and 2015 (**Figure 4A**). These keywords are in line with our previous analysis of publication volumes. A crucial guidance document in this field was published in 2011, leading to increased literature in this field since then (2, 15). Moreover, deep learning and radiomics have become increasingly popular over the past 3 years (22–24). These findings suggest that artificial intelligence to diagnose pulmonary nodules is the recent focus for GGO.

In total, 141 keywords were divided into five clusters using keyword co-occurrence analysis, where each cluster represented a major research topic associated with GGO. The largest group (red) was associated with prognosis and diagnosis. Low-dose CT was the primary method for screening and follow-up of patients with pulmonary nodules. CT can provide high spatial and temporal resolutions and high-resolution contrast images of chest anatomy (25). Although the radiation dose of low-dose CT is considered safe, a small risk of inducing cancer exists. In addition, the new ACCP guidelines recommend less frequent testing for indolent tumors, such as GGO (26). Group 2 (green) was associated with artificial intelligence. On CT, the imaging features of early lung adenocarcinoma and benign GGO were significantly similar. Hence, benign and malignant GGO classification is challenging. The observation of lung nodules is labor-intensive and time-consuming, resulting in varied individual differences. Hence, different computer-aided diagnostic models are proposed based on CT images to quickly and accurately predict the malignant degree of GGO (27). Two architectures of this model involve radiomic feature-based and deep learning-based schemes (28, 29). Although many studies combine image analysis and artificial intelligence to predict benign and malignant GGO in the lungs, no consensus has been reached yet (30, 31). Hence, further validation will require multicenter, high-quality data sets, and prospective randomized controlled trials. In group 3 (blue), treatment was the primary theme. Currently, the primary management methods for pulmonary nodules are medical follow-up and surgical resection; however, these methods are not advisable for all patients. The commonly used non-surgical treatments include systemic therapy, stereotactic body radiation therapy, and image-guided thermal ablation (32, 33). Group 4 (yellow) is associated with preoperative positioning and minimally invasive surgery. Surgery is the treatment for patients prospectively diagnosed with malignant GGO. Lobectomy and systemic lymph node dissection are standard treatments for lung cancer surgery. In recent years, sublobectomy showed a better therapeutic effect for some patients with early lung cancer with pulmonary nodules (34, 35). Therefore, doctors should preserve healthy lung tissues to ensure the therapeutic effect, reducing the prevalence of surgery and improving the quality of life after surgery. At present, the choice of sublobectomy has no consensus. The size, location, and proportion of solid components of pulmonary nodules should be considered to decide the surgical method (35, 36). Group 5 (purple), the last one, was associated with GGO pathology. The persistence of GGO nodules indicates atypical adenomatous hyperplasia, adenocarcinoma in situ, microinvasive adenocarcinoma, or invasive adenocarcinoma (37, 38).

The present study has several limitations. First, the citation analysis may not adequately measure the impact of the article. Due to the short period between publication and citation, recent articles are at a disadvantage as citations are less likely to occur. However, citations are one of the most reliable tools for measuring research quality. Second, the study did not include an article that may be a milestone in 2022, possibly affecting the analysis of the latest research hotspots. In addition, only English literature from the WoSCC was included, leading to the omission of important literature from other databases or languages. Nevertheless, WoSCC is a commonly used database for bibliometric analysis and represents most of the information to a certain extent.

## 5 Conclusions

With the increased number of patients with GGO, the management of patients with pulmonary GGO is challenging. Countries and institutions should deepen academic exchanges and conduct more cooperative research on GGO. Future research will focus on artificial intelligence diagnostic and surgical methods for GGO. Researchers are increasingly committed to accurately and quickly identifying benign and malignant GGO and achieving minimally invasive surgical treatment with less organ damage.

## Data availability statement

Publicly available datasets were analyzed in this study. This data can be found here: web of science.

## Author contributions

XL: data collection and drafting of the manuscript, GZ: drafting and revision, SG: data analysis, QX: data collection and data collection, JH: design of this work and data analysis. All authors contributed to the article and approved the submitted version. XL and GZ contributed equally to this article. All authors contributed to the article and approved the submitted version.
