# Supplementary figures and images for: Knowledge mapping visualization of the pulmonary ground-glass opacity published in the web of science
Source: Front Oncol. 2022 Dec 22;12:1075350. doi: 10.3389/fonc.2022.1075350 (PMC9815441; doi:10.3389/fonc.2022.1075350)

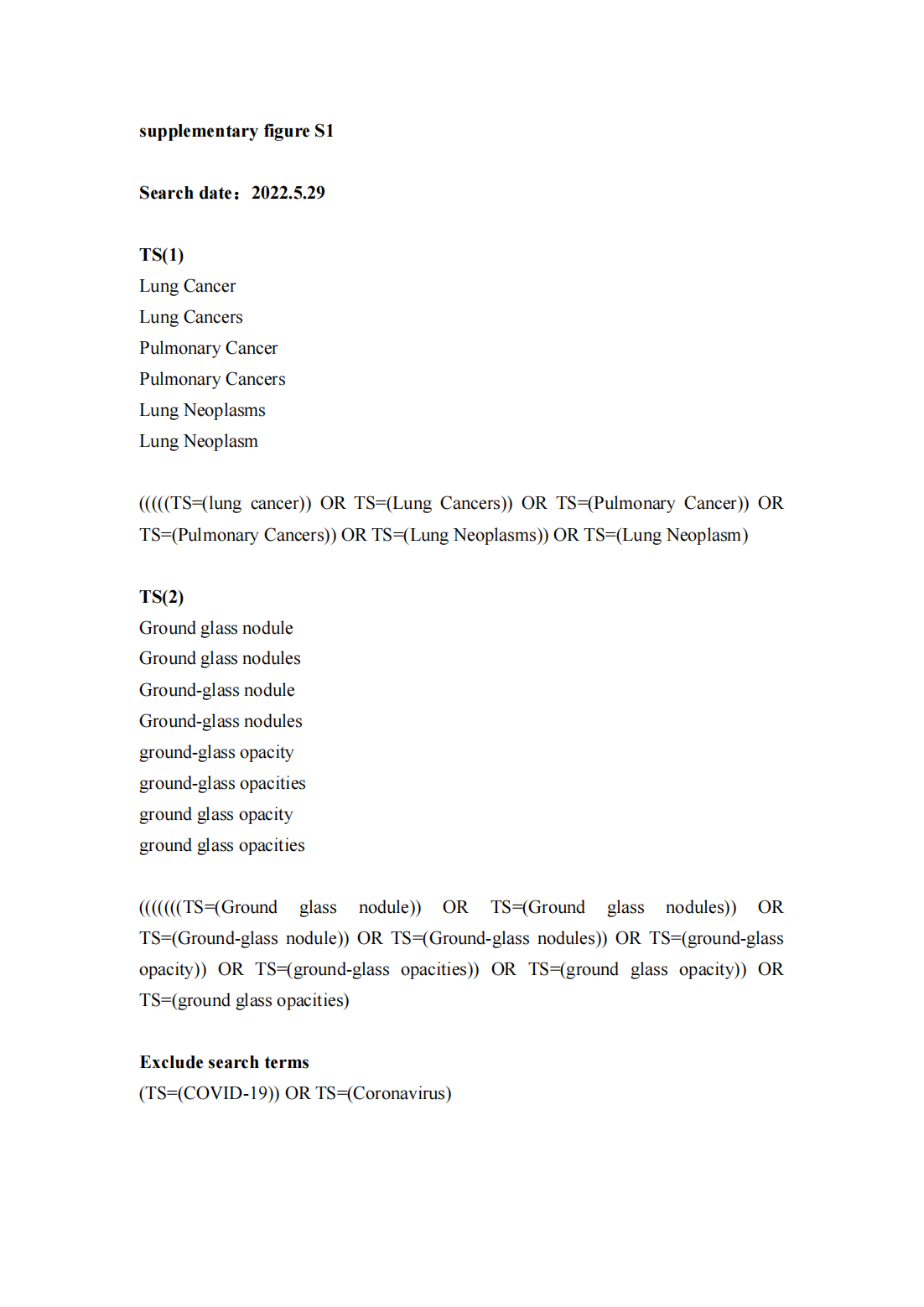

Supplement: Supplementary file 1 [file Image_1.tif]

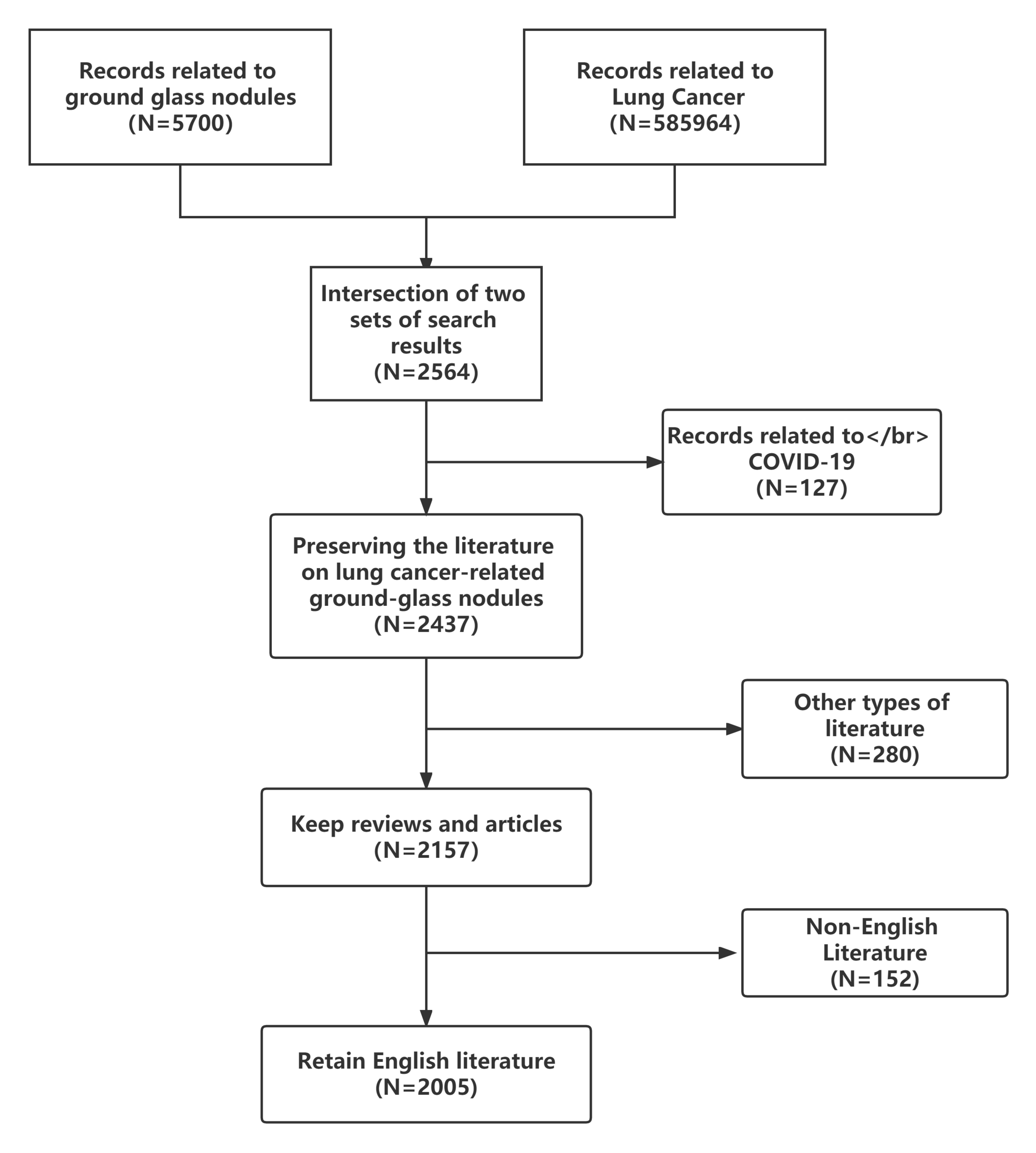

Supplement: Supplementary file 2 [file Image_2.jpg]

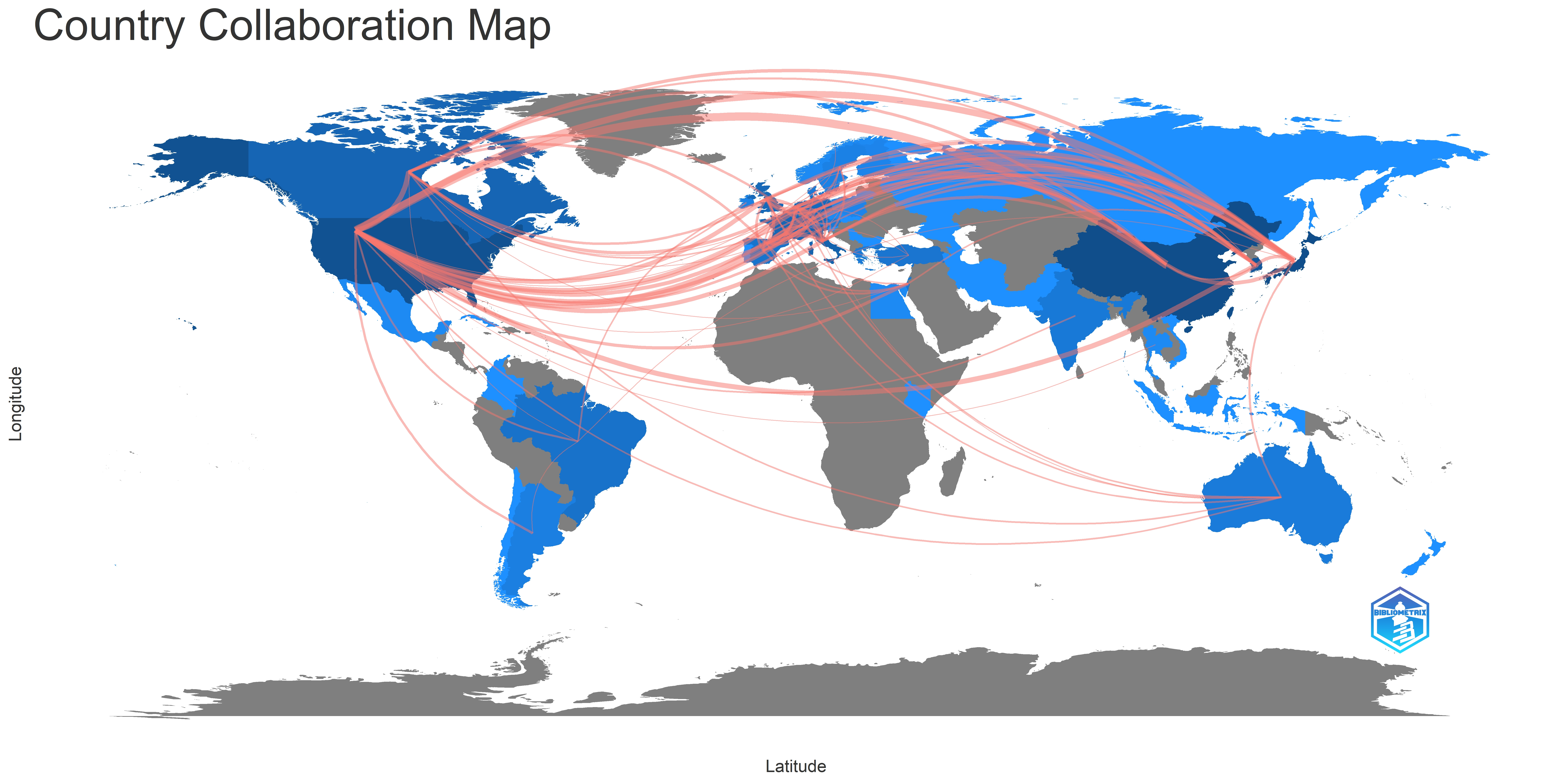

Supplement: Supplementary file 3 [file Image_3.jpg]

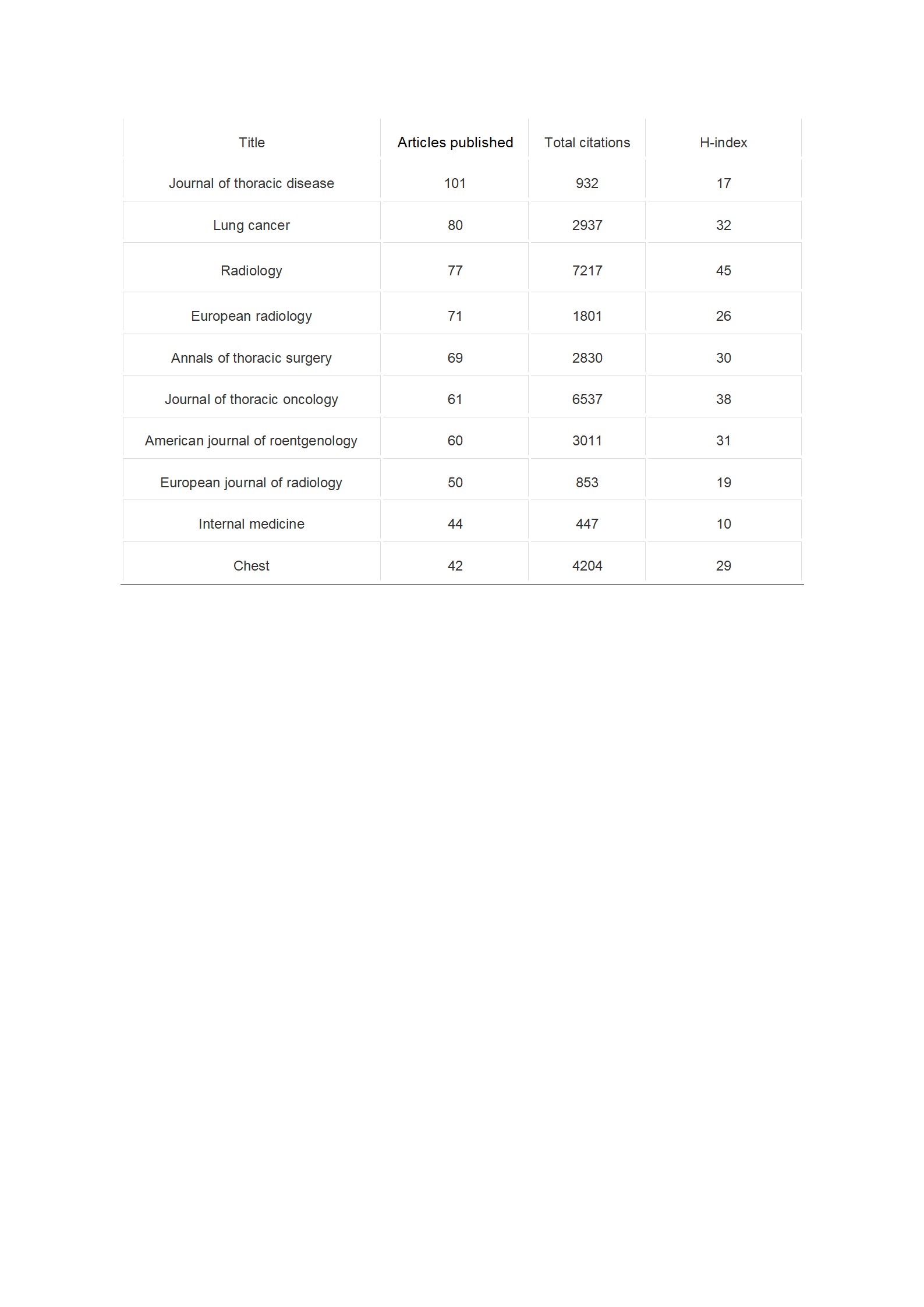

Supplement: Supplementary file 4 [file Image_4.jpg]
